# A neurodynamic model of inter-brain coupling in the gamma band

**DOI:** 10.1152/jn.00224.2022

**Published:** 2022-09-07

**Authors:** Quentin Moreau, Lena Adel, Caitriona Douglas, Ghazaleh Ranjbaran, Guillaume Dumas

**Affiliations:** ^1^Precision Psychiatry and Social Physiology Laboratory (PPSP), CHU Sainte-Justine Research Center, Montreal, Quebec, Canada; ^2^Department of Psychiatry, University of Montréal, Quebec, Canada; ^3^Integrated Program in Neuroscience, McGill University, Montreal, Quebec, Canada; ^4^Division of Social and Transcultural Psychiatry, McGill University, Montreal, Quebec, Canada; ^5^Lady Davis Institute for Medical Research, Montreal Jewish General Hospital, Montreal, Quebec, Canada; ^6^Mila—Quebec AI Institute, University of Montréal, Quebec, Canada; ^7^Human Brain and Behavior Laboratory, Center for Complex Systems and Brain Sciences, Florida Atlantic University, Boca Raton, Florida

**Keywords:** cross-frequency coupling, EEG, hyperscanning, Kuramoto model, synchronization

## Abstract

The use of EEG to simultaneously record multiple brains (i.e., hyperscanning) during social interactions has led to the discovery of inter-brain coupling (IBC). IBC is defined as the neural synchronization between people and is considered to be a marker of social interaction. IBC has previously been observed across different frequency bands, including theta [4–7 Hz]. Given the proximity of this frequency range with behavioral rhythms, models have been able to combine IBC in theta with sensorimotor coordination patterns. Interestingly, empirical EEG-hyperscanning results also report the emergence of IBC in the gamma range [>30 Hz]. Gamma oscillations’ fast and transient nature makes a direct link between gamma-IBC and other (much slower) interpersonal dynamics difficult, leaving gamma-IBC without a plausible model. However, at the intrabrain level, gamma activity is coupled with the dynamics of lower frequencies through cross-frequency coupling (CFC). This paper provides a biophysical explanation, through the simulation of neural data, for the emergence of gamma inter-brain coupling using a Kuramoto model of four oscillators divided into two separate (brain) units. By modulating both the degree of inter-brain coupling in the theta band (i.e., between-units coupling) and CFC (i.e., intraunit theta-gamma coupling), we provide a theoretical explanation of the observed gamma-IBC phenomenon in the EEG-hyperscanning literature.

**NEW & NOTEWORTHY** The last years were marked by an increasing interest in multiple-brain recordings. However, the inter-brain coupling arising across interacting individuals also sparks debates about the underlying biological mechanisms. The inter-brain coupling in the gamma band [>30 Hz] was particularly criticized for lacking a theoretical framework. Here, by using biologically informed neural simulations with the Kuramoto model, we assess the role of intra- and inter-brain neural dynamics in the emergence of inter-brain synchrony in the gamma band.

## Graphical abstract

**Figure:**
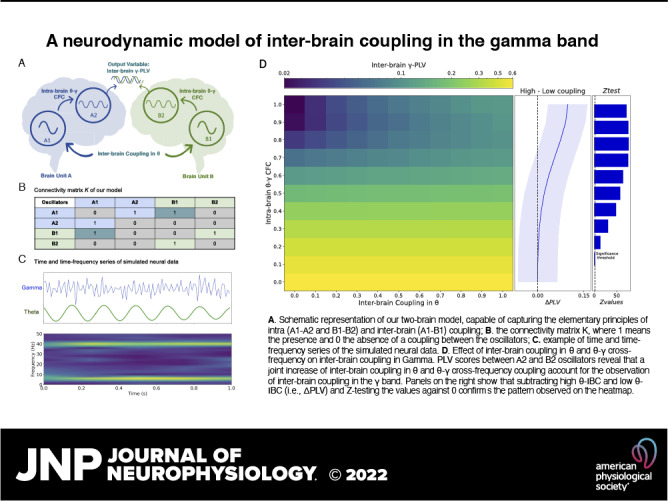

## INTRODUCTION

Social interaction is a core feature of human life. However, the neural mechanisms that support our capacity to interact with others remain poorly understood due to the fact that neuroscience has mainly focused on recording single participants in isolation rather than assessing several interacting agents simultaneously. Recently, however, the simultaneous recording of multiple brains, commonly known as hyperscanning, has become a popular method within the field of social neuroscience to study interpersonal brain dynamics (1–4). Specifically, electroencephalography (EEG) hyperscanning led to the report of a phenomenon called inter-brain coupling [IBC, but see also similar terms such as inter-brain synchrony/synchronization (3, 5, 6)], a temporal synchronization of neural signals across brains when participants interact (7–10). Inter-brain coupling is now widely accepted as a marker of social engagement and successful interpersonal communication, despite the doubt regarding its epiphenomenal nature not being completely lifted (11, 12). Furthermore, the current knowledge on IBC relies on empirical data. In this paper, we aimed at simulating EEG hyperscanning data using a simplistic model to advance our understanding of underlying phenomena captured by inter-brain coupling methods.

IBC has been mostly highlighted using phase synchrony indices such as the phase-locking value (PLV; 13), the phase-locking index (PLI; 14) and the partial directed coherence (PDC; 15). This revealed a variety of inter-brain synchronizations across different frequency bands, including in the theta (4–7 Hz) (10, 16–18) and the alpha/mu (8–13 Hz) ranges (3, 19, 20). According to the laws of coordination dynamics, behavioral rhythms of participants during an interaction can both influence and be reciprocally influenced by the behavior of the partner, resulting in a convergence of the dyad’s behavioral rhythms toward a common frequency (21, 22). Given the proximity of theta and alpha/mu frequencies with the rhythms of behavioral sensorimotor coordination, IBC in these ranges can be modeled according to the same coordination dynamics principles and reciprocal exchanges of information across members of an interaction, leading to the brain-behavior coordination dynamics framework (3, 21–24).

However, inter-brain synchronizations in higher frequencies such as in the gamma range (>30 Hz) have also been reported (3, 25–27). Gamma waves are fast and ultra-fast transient oscillations believed to support local computation (28–31). Hence, the time scale of this frequency band cannot be directly attributed to behavioral coordination rhythms, leading some to question the validity of observed gamma-IBC (12). On the other hand, at the intrabrain level, an increase of local gamma amplitude is supported by the phase of lower frequencies theta-gamma coupling) through cross-frequency coupling (CFC; 32–34). CFC has been described as a physiological mechanism capable of coordinating neural dynamics across spatial and temporal scales, where the firing of local neural populations is controlled by larger whole brain dynamics (35). Based on these characteristics, we propose that previously observed gamma IBC during social interactions can be explained by the combination of two neurophysiological occurrences: *1*) inter-brain coupling of lower frequency waves according to coordination dynamics and *2*) intrabrain level cross-frequency coupling.

## MATERIAL AND METHODS

### Dynamical Model of Gamma IBC with Kuramoto

Leveraging Python implementation of Kuramoto systems (36), we implemented our model in Python 3.7 (37) using libraries such as Numpy (38), and SciPy (39) for the computational analyses, and Matplotlib (40) for the visualization. The Kuramoto model also holds several assumptions: that all oscillators are identical, that the oscillators are innately coupled, and that the oscillations follow a sinusoidal pattern (41–44). Finally, the phase θ of an oscillator *i* at time *t* is described by the following dynamical equation:

$$\frac{{\text{dθ}}_{i}(t)}{\text{d}t}={\text{ω}}_{i}(t)+\overset{n}{\underset{j=1}{\sum }}K_{ij}\text{sin}({\text{θ}}_{j}(t)-{\text{θ}}_{i}(t)),$$

where *K_ij_* is the coupling matrix with coupling from oscillator *i* to oscillator *j* and *ω_i_* is the frequency of oscillator *i*.

**Figure 1.**
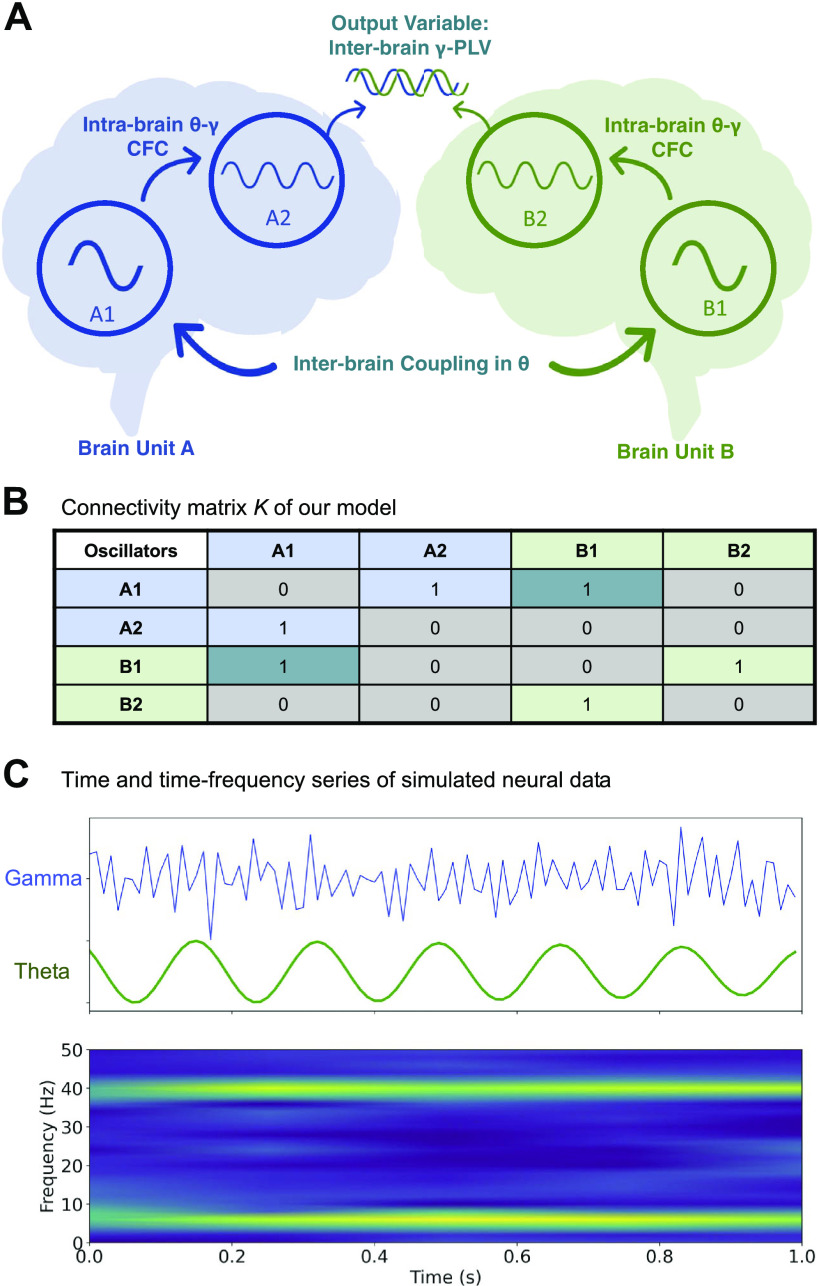
Overview of the model. *A*: schematic representation of our two-brain model, capable of capturing the elementary principles of intra- (A1-A2 and B1-B2) and inter-brain (A1-B1) coupling. *B*: the connectivity matrix *K*, where 1 means the presence and 0 the absence of a coupling between the oscillators. *C*: example of time and time-frequency series of the simulated neural data. CFC, cross-frequency coupling; PLV, phase-locking value.

As mentioned earlier, our model is composed of four oscillators, two in each brain unit (oscillators A1 and A2 in brain unit A and B1 and B2 in brain unit B, see Fig. 1). The connectivity matrix *K* is illustrated in Fig. 1*B.* The inter-brain coupling between A1 and B1 in the theta band and the intrabrain theta-gamma CFC (between A1 and A2 and B1 and B2) were programmed to range from 0 to 1, by steps of 0.1.

### Inter-brain Coupling Measure

To quantify the coupling between the A2 and B2 gamma oscillators, we used the phase locking value (i.e., PLV) which provides a frequency-specific phase synchrony measure between two signals across time (13) and is widely used in both intra- and inter-brain EEG studies (3, 24, 45, 46). We applied a Hilbert transform to extract the instantaneous phase of the signals from oscillators A2 and B2 (see Fig. 1*A*) and computed the γ-PLV via the following equation:

$${\text{PLV}}_{\text{A2,B2}}\;=\;\frac{1}{T}\left|\overset{N}{\underset{t=1}{\sum }}e^{i({\text{θ}}_{A2}(t)-{\text{θ}}_{B2}(t))}\right|,$$

where *T* is the number of sampled time points and θ_A2_(*t*) and θ_B2_(*t*) are the instantaneous phase values of oscillators B and D at time point *t*. PLV values range from 0 to 1, where 0 reflects an absence of phase synchrony and 1 an identical relative phase between the two signals.

### Signal-to-Noise Ratio

By extracting the signal and the noise amplitude of the simulated time series, we computed the signal-to-noise ratio (SNR) using the following formula:

$$\text{SNR }(dB)\;=\;20\times {\text{log}}^{10}\frac{\text{Signal}}{\text{Noise}}.$$

### Data Availability

**Figure 2.**
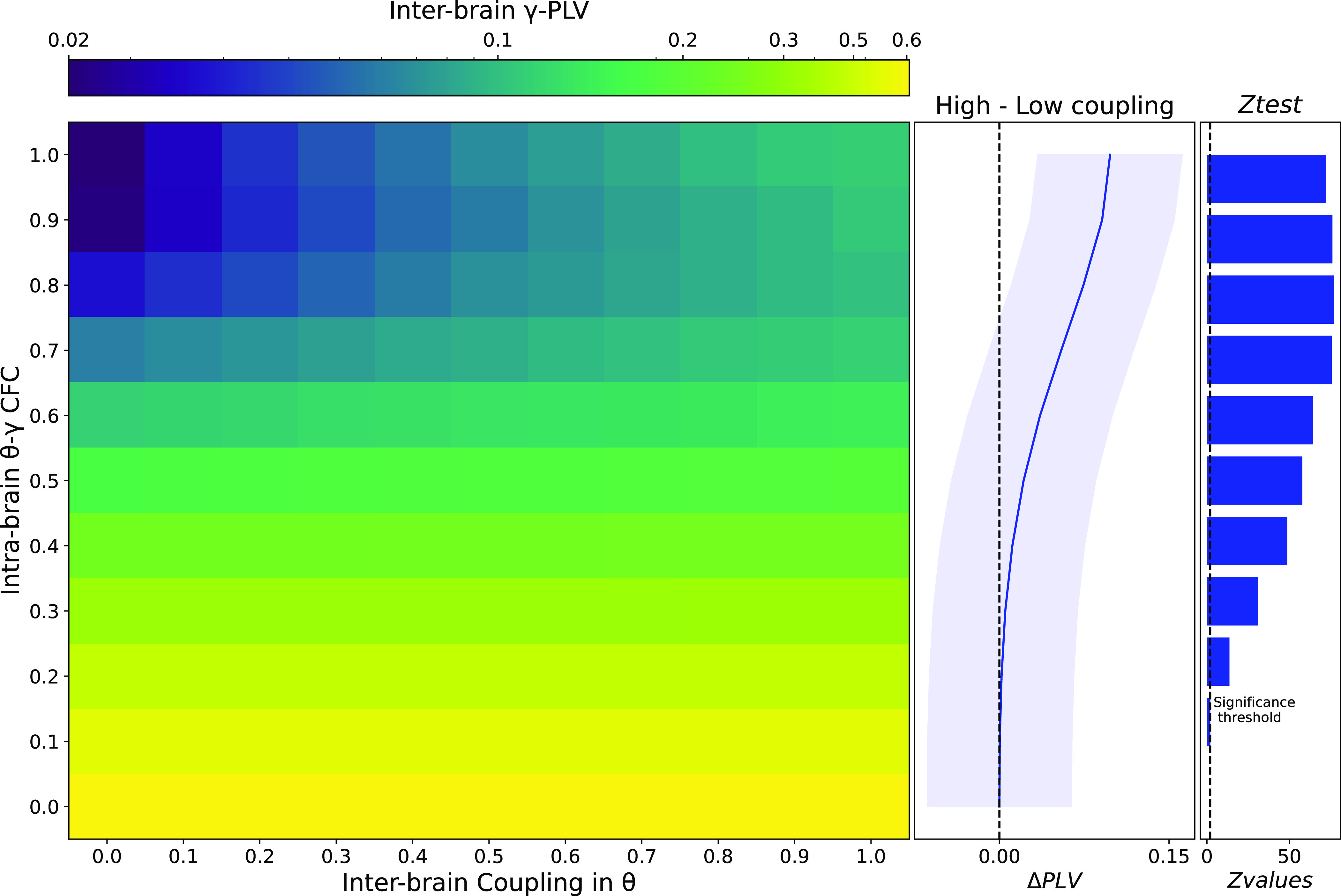
Effect of inter-brain coupling in θ and θ-γ cross-frequency on inter-brain coupling in gamma. Phase-locking value (PLV) scores between A2 and B2 oscillators reveal that a joint increase of inter-brain coupling in θ and θ-γ cross-frequency coupling account for the observation of inter-brain coupling in the γ band. Panels on the right show that subtracting high θ-inter-brain coupling and low θ-IBC (i.e., ΔPLV) and *Z*-testing the values against 0 confirms the pattern observed on the heatmap. CFC, cross-frequency coupling.

The current manuscript only relies on computational simulations, no data has been recorded. All codes are available at https://github.com/ppsp-team/Hyper-Model (archive https://doi.org/10.5281/zenodo.7047107). The data folder contains the numerical matrices generated to reproduce Fig. 2.

## RESULTS AND DISCUSSION

### Schematic Model

To test our hypothesis that IBC in gamma can be accounted for by the joint effects of theta IBC and theta-gamma CFC, we conceptualized a simple computational model of coupled oscillators simulating EEG data from two brains. We opted for a model capable of capturing the elementary principles of intra- and inter-brain coupling with minimal features. As illustrated in Fig. 1*A*, our model contains two brains, represented as two separate units (units A and B), that are coupled together through inter-brain coupling in the theta band (θ), while within each unit theta and gamma (γ) are coupled through CFC.

### Kuramoto Simulations and Signal-to-Noise Ratio

Previous studies used the Kuramoto model for weakly coupled oscillators (41, 44) to demonstrate the effect of intrabrain anatomical and functional connectivity on IBC (23), as well as interpersonal behavioral synchronization strategies and how they rely on the relationship between intra- and interunit coupling (47). Generally, the Kuramoto model describes a system of coupled oscillators where the individual oscillators are attracted and entrained to the average rate (in our case, this refers to phase convergence rather than frequency convergence (23, 42). Even though Kuramoto models do not account for the nonstationarity nature of neural data, they can still uncover essential concepts of neuro-oscillatory dynamics and explain synchronous coupling in complex systems (48). Here, we implemented our model using Kuramoto oscillators, following the connectivity matrix *K* (Fig. 1*B*). The mean frequency of the oscillators A1 and B1 was set at 6 Hz (±1, i.e., within the theta range), and the mean frequency of the oscillators A2 and B2 was set at 40 Hz (±1, i.e., within the gamma range) and without time delays (i.e., we did not include time lags in our model). We simulated time series with a length of 40 s (by steps of 10 ms). The inter-brain coupling between A1 and B1 in the θ band and the intrabrain theta-gamma CFC (between A1 and A2 and B1 and B2) were programmed to range from 0 to 1, by steps of 0.1. Simulations were run 10,000 times to obtain stable results. We applied a Gaussian noise (μ = 0, σ = 0.6), resulting in a signal-to-noise ratio of 6.575 dB, comparable with SNR found in the EEG literature (49).

### Inter-brain γ Connectivity

To estimate inter-brain connectivity between the simulated time series of oscillator A2 and B2, we computed the phase locking value (see material and methods). The γ-PLV matrix containing the inter-brain connectivity values between the oscillators A2 and B2 is illustrated by the heatmap in Fig. 2. The first observation is that constant high PLV values in gamma occur for low CFC values (i.e., between 0 and 0.2). This is shown by consistent high PLV scores along the *x*-axis on the heatmap in Fig. 2. This observation seems paradoxical, as intuitively one would expect an increase in PLV values with increasing theta IBC (i.e., a gradient from low to high PLV values along the *x*-axis). However, these PLV values can be labeled as spurious coupling, given that the constant high PLV values are simply a result of the lack of modulation in CFC strength (0–0.2). When intrabrain gamma is not modulated by theta within either of the units, the PLV measure confuses the similarity of the oscillators in each brain for inter-brain synchrony (45). Our result is a good illustration of the fact that absolute PLV values alone are not meaningful for empirical hyperscanning data, but that a careful choice of contrasting conditions (e.g., synchrony vs. nonsynchrony) is necessary to interpret IBC values correctly (50).

Our second crucial finding is that the increase of θ-γ CFC (above 0.3 on the *y*-axis of the heatmap in Fig. 2) together with an increase of inter-brain coupling in the θ band is associated with higher PLV scores (see top-right corner values of the heatmap in Fig. 2). In addition, we subtracted the values (i.e., ΔPLV) with the highest degree of IBC (i.e., inter-brain coupling in the theta band = 1) from the values with the lowest degree of coupling (i.e., inter-brain coupling in the theta band = 0) and performed a one-sample *z* test (*n* = 10,000) on ΔPLV values against 0 for each values of CFC, confirming incremental effect of CFC on γ-PLV (see bar plots in Fig. 2): *Z*_CFC_ = 0 = −0.164, *P* = 0.565; *Z*_CFC_ = 0.1 = 2.050, *P* < 0.001; *Z*_CFC_ = 0.2 = 13.657, *P* < 0.0001; *Z*_CFC_ = 0.3 = 30.943, *P* < 0.0001; *Z*_CFC_ = 0.4 = 48.661, *P* < 0.0001; *Z*_CFC_ = 0.5 = 57.844, *P* < 0.0001; *Z*_CFC_ = 0.6 = 64.354, *P* < 0.0001; *Z*_CFC_ = 0.7 = 75.685, *P* < 0.0001; *Z*_CFC_ = 0.8 = 77.022, *P* < 0.0001; *Z*_CFC_ = 0.9 = 76.077, *P* < 0.0001; *Z*_CFC_ = 1 = 72.245, *P* < 0.0001. These results highlight the impact of the joint increase of θ inter-brain coupling and θ-γ cross-frequency coupling on γ-PLV. Future empirical research in both humans (using M/EEG) and animal models (51–53) should account for these two phenomena by target θ and γ dynamics in social contexts, both at the intra- and inter-brain level. Causal relationship between γ IBC and θ-γ CFC could also be investigated through perturbation/stimulation techniques using transcranial electrical stimulation (tES) techniques (5, 54).

### Biophysical Explanation for gamma Inter-brain Coupling

Our model provides a biophysical explanation for gamma inter-brain coupling, an observation that has often been reported in human EEG hyperscanning studies. Although IBC in lower frequencies such as theta and alpha is in line with brain-behavior coordination dynamics (3, 21–24), doubts regarding the validity and the reliability of observed gamma inter-brain coupling (i.e., gamma IBC not being a neural correlate of interaction) are not completely lifted (12).

Based on evidence from intrabrain, inter-brain, and computational connectivity studies (3, 23, 47, 55), but also recent account of inter-brain correlations in animals including bats (53) and mice (51, 52), we hypothesized that gamma IBC could be explained and modeled according to two distinct neurophysiological processes, namely inter-brain coupling in theta (10, 16–18) and intrabrain theta-gamma cross-frequency coupling (33, 34). First, we showed that our 4-oscillator Kuramoto model, divided into two separate units, was able to replicate the core characteristics of both CFC and IBC. Furthermore, our model confirms the hypothesis that IBC in gamma can be ascribed to intrabrain theta-gamma cross-frequency and theta inter-brain coupling, by showing higher PLV scores during the joint increase of both parameters. Hence, our simulations give a biophysical model to the observed gamma-IBC in the EEG-hyperscanning literature (3, 25–27). Beyond supporting the empirical observations of gamma-IBC, this model also nuances the claim about their epiphenomenalism (12). Indeed, although the causal link is not directly carried by gamma frequency exchange of information, gamma rhythms associated with intra and inter-brain processes can statistically be synchronized during social interaction and thus lead to Hebbian learning in networks associated with self and other behavior (56), thus developing mirroring in related brain structure (57, 58). Our results also illustrate the importance of taking into account both intra- and inter-brain factors in the design, analysis, and interpretation of hyperscanning experiments.

Altogether, through computational modeling, our approach and results advance our mechanistic understanding of IBC, which is crucial to reaching a coherent theoretical framework describing causal relations between socio-cognitive factors, behavioral dynamics, and neural mechanisms involved in multi-brain neuroscience (59).

## GRANTS

This study was supported by the Institute for Data Valorization, Montreal (IVADO; CF00137433). G.D.’s salary was covered by the Fonds de recherche du Québec (FRQ; 285289). L.A. was funded by the Foundation Mindstrong (2021) of the Jewish General Hospital in Montréal. This study was enabled in part by support provided by Calcul Québec (www.calculquebec.ca) and Digital Research Alliance of Canada (www.alliancecan.ca).

## DISCLOSURES

No conflicts of interest, financial or otherwise, are declared by the authors.

## AUTHOR CONTRIBUTIONS

G.D. conceived and designed research; Q.M., L.A., and G.D. analyzed data; Q.M., L.A., and G.D. interpreted results of experiments; Q.M., G.R., and G.D. prepared figures; Q.M., L.A., C.D., and G.D. drafted manuscript; Q.M., L.A., C.D., G.R., and G.D. edited and revised manuscript; Q.M., L.A., C.D., G.R., and G.D. approved final version of manuscript.
